# Patient preferences for analgesia in lung surgery: an observational cohort study

**DOI:** 10.1186/s12871-025-03448-6

**Published:** 2025-11-17

**Authors:** Louisa N. Spaans, M. Elske van den Akker-van Marle, Martijn van Dorp, Joël van der Niet, Erik M. von Meyenfeldt, Hendrik H.L. Jiang, R. Arthur Bouwman, Ad F.T.M. Verhagen, Marcel G.W. Dijkgraaf, Frank J.C. van den Broek

**Affiliations:** 1 Department of Surgery, Máxima MC, Po box 7777, Veldhoven, MB 5500 The Netherlands; 2 Amsterdam UMC location University of Amsterdam, Epidemiology and Data Science, PO BOX 22700, Amsterdam, DE 1100 The Netherlands; 3 Department of Biomedical Data Sciences, Unit Medical Decision Making, LUMC, Leiden, 2300 RC The Netherlands; 4 Department of Cardiothoracic Surgery, Amsterdam UMC location Vrije Universiteit Amsterdam, De Boelelaan 1117, Amsterdam, HV 1081 The Netherlands; 5 Cancer Center Amsterdam, Cancer Treatment and Quality of Life, De Boelelaan 1117, Amsterdam, HV 1081 The Netherlands; 6 Department of Surgery, Elisabeth Tweesteden Ziekenhuis Hilvarenbeekse Weg 60, Tilburg, GC 5022 The Netherlands; 7 Department of Surgery, Albert Schweitzer Hospital, Albert Schweitzerplaats 25, Dordrecht, AT 3318 The Netherlands; 8 Department of Surgery, Laurentius Hospital Hoofdingang via de Oranjelaan, Monseigneur Driessenstraat 6, Roermond, 6043 CV The Netherlands; 9 Department of Anesthesiology and Pain Medicine, Catharina Hospital, Po box 1350, Eindhoven, 5602 ZA The Netherlands; 10 Department of Electrical Engineering, Signal Processing Systems, Eindhoven Technical University, Po box 513, Eindhoven, MB 5600 The Netherlands; 11 Department of Cardio-thoracic Surgery, Radboud University Medical Center, Po box 9101, Nijmegen, 6500 HB The Netherlands; 12 Epidemiology and Data Science, Methodology, Amsterdam UMC location University of Amsterdam, Amsterdam Public Health, Po box 22700, Amsterdam, 1100 DE The Netherlands

**Keywords:** Patient preferences, Pain, Guidelines, Thoracic surgery, ERATS, VATS

## Abstract

**Background:**

Optimal analgesia following thoracoscopic lung surgery is crucial for patient comfort and effective recovery. Despite the lack of high quality evidence in guidelines, experts favour locoregional analgesic techniques above thoracic epidural analgesia (TEA), without considering patient preferences. This study investigates patient choices to aid in shared-decision making and incorporation in guidelines.

**Methods:**

Through adaptive conjoint analysis (ACA) concerning attributes (characteristics) related to analgesic techniques and treatment trade-off methods (TTM) comparing scenarios with locoregional analgesia against TEA, 200 patients planned for thoracoscopic lung resection in five Dutch hospitals received online questionnaires. In the TTM, patients were repeatedly asked to choose between two scenarios: one describing thoracic epidural analgesia with fixed low levels of pain and the other representing locoregional analgesia with varying pain levels, to assess trade-off thresholds. For the ACA, Relative Importance (RI) of the characteristics was calculated with 95%-confidence intervals (CI).

**Results:**

Response rates of ACA and TTM questionnaires were 72% (144/200) and 71% (141/200) respectively. The most important characteristics were state of consciousness (‘awake or under general anaesthesia’) while receiving the analgesic technique (RI 20.45; 95%-CI 19.12–21.75) and mobilisation (RI 19.42; 95%-CI 18.45–20.38). In the TTM, 10 patients (7.1%) consistently chose the TEA scenario, irrespective of the benefits of locoregional analgesia. In contrast, 131 patients (92.9%) preferred experiencing more moments of pain as trade-off for the potential advantages associated with locoregional analgesia.

**Conclusion:**

Regarding analgesia following thoracoscopic lung surgery, patients considered the state of consciousness while receiving the analgesic technique (awake or under general anaesthesia) and postoperative mobility as the most important characteristics. Over 92% of patients are willing to accept more moments of pain as trade-off for the potential benefits of locoregional analgesia. These findings are aligned with current guideline recommendations and support the inclusion of patient preferences in shared decision-making.

**Supplementary Information:**

The online version contains supplementary material available at 10.1186/s12871-025-03448-6.

## Introduction

Thoracic surgery is known to be among the most painful surgical procedures. Despite advancements in minimally invasive techniques, managing postoperative pain following thoracoscopic lung surgery continues to be a significant challenge [1]. It is important to acknowledge that optimal postoperative pain control is crucial for patient comfort, early mobilization, prevention of complications, and hence successful recovery, which is in line with enhanced recovery after thoracic surgery (ERATS) guidelines.

In addition to basic systemic analgesics, locoregional analgesic techniques are recommended to reduce opioid consumption and further enhance recovery. Various (loco)regional techniques are available, but the trade-off between their potential benefits and drawbacks may differ between healthcare professionals and patients [1–3]. While thoracic epidural analgesia (TEA) is valued for its optimal pain relief, critics highlight its adverse effects leading to the discouragement of TEA in recent PROSPECT guidelines despite the lack of high level of evidence on this topic [4, 5]. Rispoli et al. suggest that non-neuraxial blocks may offer similar analgesic efficacy to TEA while minimizing risks, supporting their increased use in thoracic surgery [6]. A recent randomized controlled trial showed that in the context of thoracoscopic lung resection, single-shot intercostal nerve block is non-inferior to TEA in terms of pain, and the additional benefits of less opioid consumption, better mobility and shorter hospitalization, position it as a viable alternative to TEA [7]. There is no evidence supporting one ‘best’ technique, thus it remains a balance between pain relief and potential adverse effects of the various (loco)regional analgesic techniques. This may be weighed differently from one patient to another, or between patients and clinicians. In an era of shared decision-making, patient preferences should merit greater consideration.

Whereas guidelines aid clinicians to promote quality of care and reduce practice variation through evidence-based recommendations, they often do not acknowledge the preference-sensitive nature of patient treatment decisions. A recent study on patient preferences in clinical guidelines showed that patient preferences were not even included in the search strategy [8]. However, clinicians do believe that patient preferences should be included in clinical-decision making due to its positive impact on quality of life and patient satisfaction.

The present study aims to investigate patient preferences concerning characteristics related to analgesic techniques following thoracoscopic lung surgery through adaptive conjoint analysis (ACA) and treatment trade-off methods (TTM). These findings can be used to assist healthcare professionals in selecting the most suitable analgesic technique during shared decision-making and assist in adding patient preferences to future guidelines.

## Methods

### Participants

This study was reported following the Strengthening the Reporting of Observational Studies in Epidemiology (STROBE) guidelines (Supplemental Table S1). According to the review of the Medical Ethics Committee Máxima MC, the rules laid down in the ‘Medical Research Involving Human Subjects Act’ (Supplemental S2) did not apply to this research proposal (Clinical trial number: not applicable). Consecutive patients undergoing thoracoscopic wedge or anatomical lung resection were asked to participate in this study through informed consent. Recruitment was done in five Dutch hospitals (one academic and four non-academic hospitals) between October 22nd 2021 and May 10th 2024. Patients participating in parallel studies were not invited to avoid an excessive burden.

### Study design

Using Sawtooth Software Lighthouse Studio version 9.8.0, internet-based questionnaires were developed based on ACA and TTM. ACA estimates the importance of different attributes based on patient trade-offs. TTM determines how much compromise a patient is willing to accept between competing outcomes.The questionnaires consisted of an informed consent which was mandatory to fill in before completing the questionnaire, demographics, and the ACA and TTM inquiries. Questionnaires were distributed via e-mail and completed prior to surgery to avoid bias due to experience with involved perioperative pain and analgesic techniques.

### Selection process of attributes

Attributes are characteristics or features. Based on a meta-analysis regarding pain management after thoracoscopic lung resection [3], attributes of analgesic techniques were listed. This list was submitted for evaluation to five thoracic surgeons and three anaesthesiologists from three Dutch hospitals (one academic) to add on possible attributes and rank them by importance from an expert point-of-view (Supplemental Table S3). The four most important attributes scored by specialists were pain reduction, pain score during coughing, postoperative nausea and vomiting (PONV) and cost. The same list was used for semi-structured interviews with seven patients from the Dutch lung cancer patient association (*Longkanker* Nederland). These patients underwent prior lung resection and thus had experience with postoperative pain and analgesia. The attributes were kept general and were not clustered into specific types of analgesia.

Four attributes related to the different analgesic techniques were chosen based on expert and patient opinions: moments of pain [1], PONV as side-effect [2], mobilisation [3] and the use of a bladder catheter [4]. To enhance the distinction between different analgesic techniques, two additional attributes were added: [5] whether the analgesic technique was executed under general anesthesia or while the patient is awake, and [6] the postoperative presence of a perineural catheter for continuous pain treatment.

Each attribute was categorized with different levels to cover the complete range of actual values regarding various analgesic techniques. The levels expressed in the questionnaires are shown in Table 1.

**Table 1:** Description of the selected attributes and their levels

#	Attribute	Levels
1	Moments of pain	3 out of 20 (15%)
		5 out of 20 (25%)
		7 out of 20 (35%)
2	Risk of nausea and vomiting as side-effect	13 out of 100 (13%)
		17 out of 100 (17%)
		21 out of 100 (21%)
3	Mobilisation	Sitting on the edge of the bed
		Walking in the patient room
		Walking freely everywhere
4	Placement of a bladder catheter	Yes
		No
5	Execution of the analgesic technique	While awake
		Under general anaesthesia
6	Presence of a perineural catheter for pain treatment	Yes
		No

### Attributes and their levels

#### Moments of pain

Inadequate pain control prompts health care workers to act, which is the case if patients provide a pain score on a numerical rating scale (NRS) ≥ 4 [9]. Based on prior research [7, 10], inadequate pain control (NRS ≥ 4) was present in 17% of pain score measurements following TEA, whereas this percentage was 21% for continuous locoregional analgesia. To provide a complete range of possible values (including single-shot analgesia), moments of pain were classified into three different levels: 15%, 25% and 35%.

#### Risk of side-effect nausea and vomiting

 PONV represents a frequently encountered side-effect of analgesics and anaesthetics. Based on previous research [10–13] approximately 21% of patients receiving TEA suffer from PONV versus 13% in case of locoregional analgesia. We therefore classified PONV into three different levels: 21%, 17% and 13%.

#### Mobilisation

 Early mobilisation is strongly recommended by the ERATS guideline [14] and was classified into: sitting on the edge of the bed, walking inside the patient room, and walking freely everywhere.

#### Use of a bladder catheter

 Analgesic techniques may be associated with the need for a bladder catheter due to urinary retention. This attribute is classified as ‘yes’ or ‘no’.

#### State of consciousness during execution of the analgesic technique

 Analgesic techniques can be employed prior to (while awake) or during surgery (under general anaesthesia). This attribute was therefore classified as ‘awake’ or ‘under general anaesthesia’.

#### Presence of a perineural catheter for pain treatment

 Local anaesthetics can be administered continuously through a (perineurally) placed catheter or by means of a single-shot injection. Therefore, this attribute was classified as ‘yes’ or ‘no’.

### Adaptive conjoint analysis (ACA)

The ACA was used as a quantitative method to elicit which attributes determined the patient’s preferences, without asking them directly which analgesic technique they preferred. The questionnaire started by asking the participants to assign the attributes a level of importance on a 4-point Likert scale. Based on their initial answers, the program generated patient-tailored pairwise scenarios (incorporating two to four attributes in random order) from which patients had to choose their preference. Herewith patients traded between these attributes, by rating at which level of one attribute they gave up for an increase in another. Depending on the preferences for a given attribute level, we were able to both determine the part-worth utility (PWU) as well as the relative importance (RI) of the attributes.

### Treatment trade-off method (TTM)

The TTM was used to determine the chosen trade-off threshold beyond which the expected benefits no longer outweigh the harmful effects on a particular analgesic technique. Pairwise scenarios were presented (Table 2), mirroring an analgesic scenario with TEA versus single-shot locoregional analgesia. The maximum number of moments of pain, beyond which the advantages of locoregional analgesia were no longer accepted, was determined by increasing the number of moments of pain in the locoregional group until the patient was triggered to choose the TEA scenario. This threshold represents the patient’s non-inferiority margin for pain, above which the potential benefits of locoregional analgesic techniques no longer justify the level of pain compared to TEA. To ensure comprehension, the TTM started with an identical number of moments of pain (3 out of 20, i.e. 15%) in the paired scenarios, as patients were expected to choose the scenario with locoregional analgesia due to its inherent advantages. Subsequently, a “ping-pong” method was employed allowing participants to trade more moments of pain for the perceived benefits of locoregional analgesia compared to TEA (Table 2).

**Table 2:** Description of the treatment trade-off method

Scenario 1: Thoracic Epidural Analgesia	Scenario 2: Locoregional Analgesia
3 out of 20 moments of pain	3* out of 20 moments of pain
21 out of 100 postoperative nausea and vomiting	13 out of 100 postoperative nausea and vomiting
Sitting on the edge of the bed	Walking outside of the patient room
Presence of a bladder catheter	Absence of a bladder catheter
Awake placement analgesic technique	Under general anaesthesia during placement analgesic technique
Presence of a perineural catheter	Absence of a perineural catheter

^*^Scenario 2 starts with the same amount of moments of pain, and was changed systematically until reaching a maximum of 20 out of 20 moments of pain

### Outcomes

The PWU of the different attribute levels and the RI of the attributes are the quantified outcome measures of the ACA. The PWU represents the relative desirability of attribute levels, with higher values indicating stronger preference and lower (or negative) values reflecting lower desirability. The RI is derived from the range of PWUs within each attribute, expressing the proportional impact of each attribute on decision-making. Attributes with a higher RI had a greater influence on patient choices.

The outcome of the TTM is the distribution of the maximum number of moments of pain beyond which the advantages related to locoregional analgesia are no longer preferred.

### Data analysis

Hierarchical Bayes estimation was used to calculate the RI of each attribute for each respondent, according to the choices made in the ACA (using the maximum difference in the average overall parth-worth utility between levels). The RI scores are normalized to sum to 100%, allowing for direct comparison of attribute influence. The RI was averaged over all respondents, providing the order of the most important attributes influencing the patient’s decision-making regarding which analgesic technique to prefer following thoracoscopic lung resection. Descriptive data was presented as means (with standard deviation (SD)). Categorical data was presented as counts and percentages (with 95% confidence intervals (CI)). Categorical outcomes were compared among groups by using the Chi-squared test. In case of zero cell frequencies, Fisher’s exact test was performed. Numerical outcomes were compared among groups by using the paired T-test. All calculations and statistical analysis were performed by using the Sawtooth Software Lighthouse Studio 9.6.1 (Sawtooth Software, Inc., Sequim, WA, USA) and the Statistical Package for Social Sciences, version 22.0 (SPSS Inc., Chicago, IL, USA).

## Results

The questionnaire was sent to 200 eligible patients who were scheduled for thoracoscopic lung resection, of which 144 completed the ACA and 141 completed the TTM questionnaire (response rates 72% and 71%, respectively). The mean age was 65.4 years (SD 9.1), and 51% were female. A total of 103 participants (72%) had undergone previous surgery and therefore had experience with postoperative analgesia. Additionally, 40 participants (28%) were experiencing pain at the time of completing the questionnaire and were using painkillers (Table 3).

**Table 3:** Patient baseline characteristics

Characteristics	Frequency
Age (mean, SD)	65.9 (9.1)
Gender (female, no. [%])	74 (51.4)
Previous surgery and experience with postoperative analgesia (no. [%])	103 (71.5)
Chronic pain (no. [%])	40 (27.8)
Currently pain complaints (no. [%])	51 (35.4)
Use of painkillers (no. [%])	39 (27.1)

### Relative importance of attributes

The most important attribute selected by patients was whether the analgesic technique was executed while awake or under general anaesthesia (RI 20.44; 95% CI 19.12–21.75), followed by mobilisation (RI 19.42; 95% CI 18.45–20.38), moments of pain (RI 16.91; 95% CI 16.03–17.78), placement of a bladder catheter (RI 16.45; 95%CI 15.44–17.45), risk of PONV (RI 14.70; 95% CI 13.86–15.54) and placement of a perineural catheter for pain treatment (RI 12.09; 95% CI 11.28–12.90) (Table 4). The comparison of the first two attributes was not statistically significant (*p* = 0.22), while these two attributes were significantly more important compared to all other attributes (*p* < 0.001). The risk of PONV and the placement of a perineural catheter for pain treatment were considered significantly less important compared to all other attributes (*p* = 0.009 and *p* < 0.001, respectively).

**Table 4:** Average Part-Worth utility and average relative importance of the attributes based on the adaptive conjoint analysis

#	Attribute	Levels	Average PWU (SD)	Average RI (95%-CI)
1	Moments of pain	3 out of 20 (15%)	50.12 (14.84)	16.91 (16.03–17.78)
		5 out of 20 (25%)	1.20 (4.63)	
		7 out of 20 (35%)	−51.32 (17.41)	
2	Risk of side-effects of nausea and vomiting	13 out of 100 (13%)	44.35 (15.18)	14.70 (13.86–15.54)
		17 out of 100 (17%)	−0.47 (3.34)	
		21 out of 100 (21%)	−43.88 (15.86)	
3	Mobilisation	Sitting on the edge of the bed	−60.19 (18.24)	19.42 (18.45–20.38)
		Walking in the patient room	3.89 (4.77)	
		Walking freely everywhere	56.30 (17.48)	
4	Placement of a bladder catheter	Yes	−49.34 (18.45)	16.45 (15.44–17.45)
		No	49.34 (18.45)	
5	Execution of the analgesic technique	While awake	−61.31 (24.19)	20.44 (19.12–21.75)
		During general anaesthesia	61.31 (24.19)	
6	Placement of a perineural catheter for pain treatment	Yes	−36.27 (14.91)	12.09 (11.28–12.90)
		No	36.27 (14.91)	

### Treatment trade-off method

**Fig. 1:**
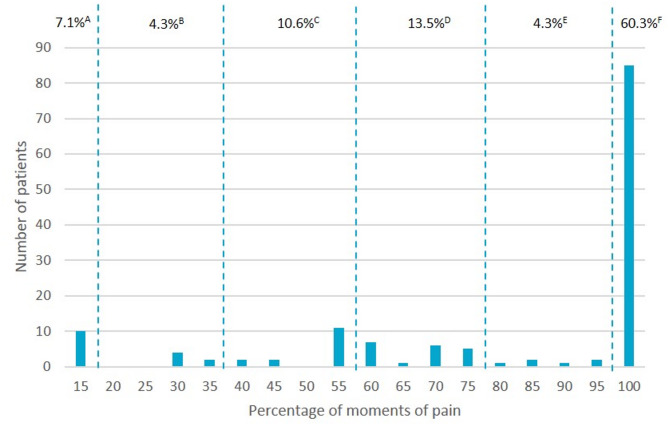
Treatment trade-off method determining the maximum moments of pain (as percentage out of 20 pain score assessments) to accept the benefits of loco regional analgesia. **A** percentage of patients choosing 15% of moments of pain (i.e. reference number of moments of pain associated with TEA). **B** incremental percentage of patients choosing 20–35% moments of pain. **C** incremental percentage of patients choosing 40–55% moments of pain. **D** incremental percentage of patients choosing 60–75% moments of pain. E: incremental percentage of patients choosing 80–95% moments of pain. **D** percentage of patients choosing 100% moments of pain 15% of moments of pain corresponds to accepting 3 out of 20 pain score assessments, whereas 100% corresponds to 20 out of 20 assessments

The maximum number of moments of pain patients were willing to tolerate to benefit from locoregional analgesia varied. Ten patients (7.1%) always chose the TEA scenario, also with identical moments of pain and regardless of the potential benefits of locoregional analgesia. In contrast, 131 patients (92.9%) preferred experiencing more moments of pain in exchange for the advantages of locoregional analgesia and 85 patients (60.3%) even accepted the maximum number of moments of pain (Fig. 1).

## Discussion (1068/1500)

Our study investigates patients’ preferences regarding postoperative pain management after thoracoscopic lung surgery. Patients chose state of consciousness (awake or under general anaesthesia) during execution of the analgesic technique and postoperative mobility as the most important attributes and the risk of PONV and the presence of a perineural catheter for pain management as the least important attributes. Interestingly, when exposing patients to two different treatment scenarios comparing TEA and single-shot locoregional analgesia, over 90% chooses having more moments of pain and 60% even accepts having pain at all moments (i.e. 20 out of 20 pain score assessments) to receive the benefits of locoregional analgesia, while only 7% always opted for the TEA scenario (i.e. even with identical moments of pain).

Current guidelines on postoperative pain management after thoracic surgery incline to locoregional analgesia due to its valued benefits which are in line with ERATS principles [5, 14]. However, the PROSPECT guideline weighs pros and cons from the perspective of experts utilizing a Delphi method and does not considerate patients’ preferences. Including patients’ opinion in guidelines makes them also more accessible to them [8]. In this way, patients can have a more active role in decision-making. In guidelines with uncertain recommendations from a medical perspective due to lack of robust data or due to small differences, health care workers should communicate this uncertainty with the patient and leave room for patients’ preferences in decision-making, even if they go against the generally accepted treatment modality [8]. Interestingly, our expert panel of five thoracic surgeons and three anaesthesiologists depicted ‘pain’ and ‘risk of PONV’ as most important attributes from their perspective, while patients chose ‘state of consciousness during execution of the analgesic technique’ and ‘postoperative mobility’ as most important, which demonstrates their discrepant points-of-view.

Patient preferences regarding analgesic techniques after thoracic surgery have not yet been reported. A systematic review on patient preferences for chronic musculoskeletal pain revealed ‘capacity to realize daily life activities’ and ‘risk of adverse events’ as most important attributes through discrete choice experiments [15]. The attribute ‘capacity to realize daily life activities’ is closely related to our key attribute ‘mobility’, both of which fall within the domain of functional ability. The choice for being more mobile aligns with the clinical benefits of ERATS and hence also covers the desire of clinical experts. However, ‘risk of PONV as adverse event’ was considered less important in our study, possibly because we included a surgical population. In the perioperative setting, the domain ‘anxiety’ seems more relevant since the execution of an analgesic technique ‘while awake or under general anaesthesia’ emerged as the most important attribute which supports the theory that patients experience the awake placement of TEA as stressful.

The willingness to trade-off acute pain relief for reduced risk of adverse effects was investigated in a preference study following abdominal surgery, and demonstrated variability in treatment choices dependent on the type and severity of adverse effects [2]. In that study, trade-offs were different for nausea or vomiting, but both had low utility scores, indicating low importance of PONV. Although we included risk of PONV with different levels of incidence in our study, patients found it significantly less relevant compared to our other attributes. We found alternative benefits related to locoregional analgesia to be more important for patients (e.g. unconscious state during administration, more postoperative mobility and absence of a bladder catheter). The present study shows that the trade-off limit set by patients is high, with even 60% of patients setting the trade-off limits at the highest number of moments of pain (i.e. 100%), corresponding to always accept the advantages of locoregional analgesia irrespective of pain. This likely reflects a strong aversion to certain procedural aspects (e.g. being awake during placement) rather than rejection of regional anaesthesia itself and prioritization of mobility and unconscious administration over absolute pain control. The recently published OPtriAL study [7] reported a modest increase in pain moments and therefore clinical non-inferiority of single-shot intercostal nerve blocks compared to TEA (8% difference). This small clinical difference aligns with patients’ willingness to trade off the benefits of locoregional analgesia for a small increase in moments of pain. This information is of great importance, since adherence to ERATS protocols is now also supported by patient preference outcome data. However, we recognize that our results cannot be generalized to all patients. A personalized approach remains essential, since patients’ willingness to accept trade-offs between pain relief and side effects varies between patients, independently of clinical, sociodemographic, and psychological factors [16]. Also, clinician-led counselling is essential to contextualize these preferences.

We realize the complexity of patient preferences and acknowledge that the decision-making process is multifactorial. It is important to highlight the relationship between expectations and patient satisfaction. A previous study [17] investigated patient expectations regarding perioperative pain management. The study identified a significant theme: a lack of communication and education between providers and patients, which increased anxiety due to unclear expectations. The same study also showed that better preoperative education and expectation, led to increased use of non-opioid analgesic strategies. Improving patient education also improved patient-care and delivered analgesic strategies in a patient-centred approach. It is important to realize that preferences of the provider can influence preferences of the patient, and the way information is provided to the patient should be objective and leave room for personalized care. By fostering more realistic expectations through better patient education, we can take an important step in understanding patient preferences and increasing patient satisfaction.

The limitations of this study include the use of an ACA in which hypothetical scenarios were presented to investigate key attributes rather than strictly adhering to real-word possibilities. Patients were not informed in detail about the types of regional anaesthesia or long-term outcomes of untreated pain. The focus was strictly on immediate postoperative characteristics. In addition, we selected a limited number of attributes to maintain the comprehensibility of the ACA and avoid excessive complexity. The sample size allowed us to draw reliable conclusions about patient preferences; however, including more patients could have resulted in narrower 95% CI and enabled subgroup analyses (e.g. by gender or age) for a more in-depth understanding of the data. Furthermore, there was lack of knowledge about the patients’ characteristics, such as knowing their previous experience with regional anaesthesia, which would have been of added value. Lastly, regarding the outcome pain patients were asked about moments of pain (NRS ≥ 4), which does not differentiate the severity of pain.

## Conclusion

Regarding pain management after thoracoscopic lung surgery, patients designated the state of consciousness while receiving the analgesic technique (awake vs. under general anaesthesia) and postoperative mobility as most important attributes for patient preference. Also, 92.9% of patients were willing to accept more moments of pain as trade-off for the benefits of locoregional analgesia. These important findings on postoperative analgesia highlight the need to integrate patient preferences into current guidelines and use these outcomes for clinical decision-making to enhance patient satisfaction and outcomes.

## Supplementary Information

Supplementary Material 1.

## Data Availability

The datasets used and/or analysed during the current study are available from the corresponding author on reasonable request.
