# Supplementary material for: Patient preferences for analgesia in lung surgery: an observational cohort study
Source: BMC Anesthesiol. 2025 Nov 17;25:567. doi: 10.1186/s12871-025-03448-6 (PMC12625438; doi:10.1186/s12871-025-03448-6)
Supplement: Supplementary file 1 — Supplementary Material 1. [file 12871_2025_3448_MOESM1_ESM.docx]

Patient Preferences for Analgesia in Lung Surgery: Supplementals

Table S1: STROBE Statement—Checklist of items that should be included in reports of ***cohort studies***

|  | Item No | Recommendation | Page |
| --- | --- | --- | --- |
| **Title and abstract** | 1 | (*a*) Indicate the study’s design with a commonly used term in the title or the abstract | 1 |
|  |  | (*b*) Provide in the abstract an informative and balanced summary of what was done and what was found | 4 |
| Introduction | | |  |
| Background/rationale | 2 | Explain the scientific background and rationale for the investigation being reported | 5 |
| Objectives | 3 | State specific objectives, including any prespecified hypotheses | 5 |
| Methods | | |  |
| Study design | 4 | Present key elements of study design early in the paper | 6 |
| Setting | 5 | Describe the setting, locations, and relevant dates, including periods of recruitment, exposure, follow-up, and data collection | 6 |
| Participants | 6 | (*a*) Give the eligibility criteria, and the sources and methods of selection of participants. Describe methods of follow-up | 6 |
|  |  | (*b*) For matched studies, give matching criteria and number of exposed and unexposed | N/A |
| Variables | 7 | Clearly define all outcomes, exposures, predictors, potential confounders, and effect modifiers. Give diagnostic criteria, if applicable | 8 |
| Data sources/ measurement | 8* | For each variable of interest, give sources of data and details of methods of assessment (measurement). Describe comparability of assessment methods if there is more than one group | 7,8 |
| Bias | 9 | Describe any efforts to address potential sources of bias | 6 |
| Study size | 10 | Explain how the study size was arrived at | N/A |
| Quantitative variables | 11 | Explain how quantitative variables were handled in the analyses. If applicable, describe which groupings were chosen and why | 8,9 |
| Statistical methods | 12 | (*a*) Describe all statistical methods, including those used to control for confounding | 8,9 |
|  |  | (*b*) Describe any methods used to examine subgroups and interactions | N/A |
|  |  | (*c*) Explain how missing data were addressed | N/A |
|  |  | (*d*) If applicable, explain how loss to follow-up was addressed | N/A |
|  |  | (*e*) Describe any sensitivity analyses | N/A |
| Results | | |  |
| Participants | 13* | (a) Report numbers of individuals at each stage of study—eg numbers potentially eligible, examined for eligibility, confirmed eligible, included in the study, completing follow-up, and analysed | 10 |
|  |  | (b) Give reasons for non-participation at each stage | N/A |
|  |  | (c) Consider use of a flow diagram | N/A |
| Descriptive data | 14* | (a) Give characteristics of study participants (eg demographic, clinical, social) and information on exposures and potential confounders | 10 |
|  |  | (b) Indicate number of participants with missing data for each variable of interest | N/A |
|  |  | (c) Summarise follow-up time (eg, average and total amount) | N/A |
| Outcome data | 15* | Report numbers of outcome events or summary measures over time | N/A |
| Main results | 16 | (*a*) Give unadjusted estimates and, if applicable, confounder-adjusted estimates and their precision (eg, 95% confidence interval). Make clear which confounders were adjusted for and why they were included | 10 |
|  |  | (*b*) Report category boundaries when continuous variables were categorized | Table 1 and 3 |
|  |  | (*c*) If relevant, consider translating estimates of relative risk into absolute risk for a meaningful time period | N/A |
| Other analyses | 17 | Report other analyses done—eg analyses of subgroups and interactions, and sensitivity analyses | N/A |
| Discussion | | |  |
| Key results | 18 | Summarise key results with reference to study objectives | 11,12 |
| Limitations | 19 | Discuss limitations of the study, taking into account sources of potential bias or imprecision. Discuss both direction and magnitude of any potential bias | 12 |
| Interpretation | 20 | Give a cautious overall interpretation of results considering objectives, limitations, multiplicity of analyses, results from similar studies, and other relevant evidence | 11,12 |
| Generalisability | 21 | Discuss the generalisability (external validity) of the study results | 11,12 |
| Other information | | |  |
| Funding | 22 | Give the source of funding and the role of the funders for the present study and, if applicable, for the original study on which the present article is based | 14 |

* Give information separately for exposed and unexposed groups.

S2: Ethical Committee review

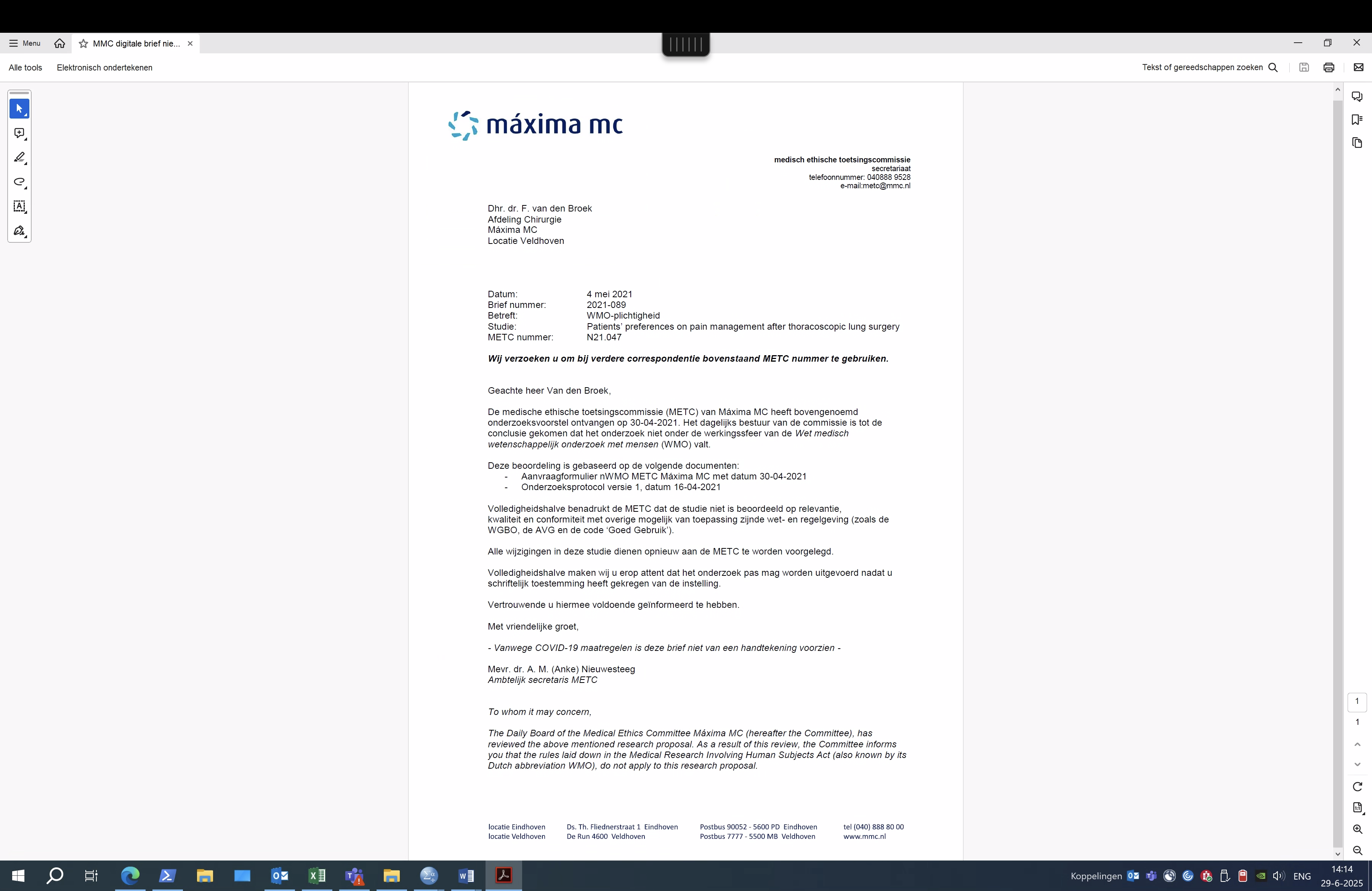


Table S3: List of outomes based on the literature and rated as important by medical specialists

| Outcome | n (%) |
| --- | --- |
| Reduction of pain | 8 (100) |
| Pain score during coughing | 8 (100) |
| Side-effects of thoracic epidural analgesia:  Nausea and vomiting | 8 (100) |
| Cost-effectiveness of the analgesic technique | 8 (100) |
| Pain score at rest | 7 (85.7) |
| Pain score during mobilisation | 7 (85.7) |
| Postoperative morfine use | 7 (85.7) |
| Side-effects of the use of opioids:  Nausea and vomiting | 7 (85.7) |
| Side-effects of thoracic epidural analgesia:  Bladder retention | 7 (85.7) |
| Mobility | 7 (85.7) |
| Patient satisfaction | 7 (85.7) |
| Presence of a bladder catheter | 7 (85.7) |
| Easy breathing | 7 (85.7) |
| A good sleep | 7 (85.7) |
| Presence of thoracic drainage | 6 (71.4) |
| Going to the toilet without assistance and taking care of personal hygiene | 6 (71.4) |
| Side-effects of morphine | 6 (71.4) |
| Length of hospital stay | 4 (57.1) |
| Awake placement of the analgesic technique | 1 (14.3) |
|  |  |
